# Efficacy of lifestyle interventions in reducing diabetes incidence in patients with impaired glucose tolerance: a systematic review of randomized controlled trials

**DOI:** 10.1186/1753-6561-6-S3-P28

**Published:** 2012-06-01

**Authors:** Uzung Yoon, Lai Lai Kwok, Athanasios Magkidis

**Affiliations:** 1 Berlin School of Public Health, Charité, Berlin, 11055, Germany; 2 Columbia University, New York, NY, 10027, USA; 3 Klinikum Mitte, Bielefeld, 33604, Germany

## Background

Every year over 3.8 million people are dying of diabetes and its complications. Lifestyle intervention was suggested to have beneficial effects in preventing and reducing diabetes incidence. Especially interventions in patients with impaired glucose tolerance (IGT), who belong to a high risk group in developing diabetes, are supposed to be very effective. According to the evidence heirarchy, a 1a level of evidence is missing and therefore a systematic review verifying the efficacy of lifestyle intervention is needed.

## Materials and methods

Systematic review: The electronic database PubMed, Embase, Cochrane Central Register of Controlled Trials, Cochrane Database of Systematic Reviews, and Health Technology Assessment database were searched. Main inclusion criteria were randomized controlled trials, impaired glucose tolerance, lifestyle intervention with control group and observation time >6 month. Outcome measures were all diabetes events, as defined by the authors of each study, all-cause mortality, diabetes mortality, and quality adjusted life years (QALY). Two independent reviewers abstracted the found studies by title, abstract and full-text analysis. Furthermore the reporting quality of each study was assessed by using the CONSORT criteria (Consolidated Standards of Reporting Trials) and the methodological quality by SIGN 50 instrument (Scottish Intercollegiate Guidelines Network methodology checklist for randomized controlled trials). The primary outcome measure was diabetes incidence. Secondary outcome measures were overall mortality, disease-specific mortality, quality adjusted life years (QALY), and clinical parameters; body mass index (BMI), weight change, blood pressure, blood parameter, smoking, alcohol consumption.

## Results

7 trials which included 25 relevant publications were identified [1-25]. Kappa Cohens for title-analysis were K=0.77, (CI=0.71-0.83), abstract-analysis K=0.81 (CI=0.64-0.92) and full-text analysis K=0.78 (CI=0.57-0.98). Overall 5663 patients were analyzed with primary follow-up time: India (3 yr), Japan (4 yr), Sweden (5 yr), Da Qing (6 yr), SIM (3 yr), DPP (5 yr), DPS (4 yr) and drop-out rate ranges from 5% to 28%. Diabetes incidence ranges from 3% to 46% in the intervention group and 9.3% to 67.7% in the control group. The India study reported ARR= 16%, RRR= 29% (p=0.018), Japan: ARR= 6.3%, RRR= 65% (p<0.001), Sweden: ARR= 4%, RRR= 25% (p=not significant), Da Qing: ARR= 22%, RRR= 32% (p<0.05), SLIM: ARR= 20%, RRR= 53% (p=0.025), DPP: ARR= 15%, RRR= 58% (significant, no p value reported), and DPS: ARR=12%, RRR= 52% (significant, no p value reported). Mortality and morbidity were only analyzed in Da Qing study which showed no statistical differences (overall mortality: HRR 0.96, CI 0.65-1.41, CVD-mortality: HRR 0.83; CI 0.48-1.40, CVD event: HRR 0.98; CI 0.71-1.37).

## Conclusions

Under consideration of heterogeneity in lifestyle interventions and follow up time of the included studies, this systematic review illustrated that lifestyle intervention can have a beneficial effect on the incidecne of diabetes in patients with impaired glucose tolerance. However, several studies found the effect of lifestyle intervention decreased after intervention was terminated. Development of standardized lifestyle intervention program is strongly needed and further long term intervention trials using this program are crucial in evidencing the long term efficacy.
